# Thirteen Nearly Complete Genome Sequences of Human Bocavirus 1 Isolated from Pediatric Inpatients in Fukushima, Japan

**DOI:** 10.1128/mra.01027-21

**Published:** 2022-01-20

**Authors:** Masatoshi Kakizaki, Yohei Kume, Reiko Suwa, Miyuki Kawase, Takashi Ono, Mina Chishiki, Sakurako Norito, Masatoki Sato, Hiroko Sakuma, Shigeo Suzuki, Mitsuaki Hosoya, Makoto Takeda, Koichi Hashimoto, Kazuya Shirato

**Affiliations:** a Department of Virology III, National Institute of Infectious Disease, Musashimurayama, Tokyo, Japan; b Department of Pediatrics, School of Medicine, Fukushima Medical University, Fukushima, Fukushima, Japan; c Hoshi General Hospital, Koriyama, Fukushima, Japan; d Ohara General Hospital, Fukushima, Fukushima, Japan; KU Leuven

## Abstract

We report 13 genomic sequences of human bocavirus 1 isolated from pediatric inpatients in Fukushima, Japan, using an air-liquid interface culture of human bronchial tracheal epithelial cells. This work suggests the endemic circulation of a human bocavirus variant with a unique amino acid signature in Fukushima.

## ANNOUNCEMENT

Human bocavirus (HBoV) belongs to the family *Parvoviridae*, with a genome consisting of approximately 5.5 kb of single-stranded DNA (ssDNA) and heterotelomeric DNA, and was first detected in Sweden in 2005 (1). HBoV has four subtypes (HBoV1 to HBoV4), and the genome contains three major open reading frames (ORFs), which encode nonstructural proteins (NSs), a nuclear phosphoprotein (NP1), and viral capsid proteins (VPs) (2). The NS ORF encodes several NSs (NS1, NS1-70, NS2, NS3, and NS4) (3). Three VPs (VP1, VP2, and VP3) are generated by the alternative splicing of VP mRNA by the NP1 protein (4). Some viruses contain putative *up1* and *ORFX* genes (5). Recent studies showed HBoV1 can cause lower respiratory tract diseases as a monoinfection and not only in combination with other respiratory pathogens, suggesting the importance of HBoV as a pathogen of respiratory infection ranked second or third after respiratory syncytial virus (6–8). Nevertheless, few full-length genomes of HBoV1 have been registered in databases; in particular, the hairpin sequences at both ends, which are necessary for viral genome replication, are often lacking (9, 10).

In this study, 13 nearly complete genome sequences of HBoV1 isolates, including hairpin sequences, were determined (Table 1). Nasopharyngeal swab specimens were collected from pediatric inpatients in Fukushima, Japan, between 2018 and 2021, and those that were HBoV1 positive by multiplex real-time PCR assays for respiratory viruses (11, 12) were used for virus isolation using an air-liquid interface culture of human bronchial tracheal epithelial cells (HBTEC-ALI) prepared as described previously (13, 14). Nucleic acids were extracted from virus stock with a QIAamp viral RNA minikit (Qiagen, Hilden, Germany) (DNA was extracted simultaneously). The libraries for next-generation sequencing were prepared using a NEBNext Ultra II RNA library preparation kit for Illumina (New England Biolabs, Ipswich, MA, USA) following the manufacturer’s instructions. Although HBoV is an ssDNA virus, indexed libraries were obtained using this kit. The indexed libraries were analyzed for 2 × 150 cycles on a DNBSEQ-G400 instrument at GENEWIZ (South Plainfield, NJ, USA). Reads were trimmed and then *de novo* assembled or mapped (based on the number of HBoV reads obtained) to the reference sequence (GenBank accession number JQ923422) using CLC Genomics Workbench v21.0.4 with default settings. The coverage of the assembled sequences was checked by mapping. The gene annotations were analyzed by VAPiD v1.6.6 (15).

**TABLE 1:** Registered HBoV1 sequences

Isolate name	Accession no.	Run data accession no.	Diagnosis	Sequence constitution method	Total no. of reads	Total no. of mapped reads	Avg coverage (×)	Length (bases)	GC content (%)	Infection type^*a*^
Fukushima_H181_2018	LC651167	DRR328227	Acute bronchitis	Assembling	14,227,470	1,009,293	26,081.54	5,371	42.17	Monoinfection
Fukushima_H216_2018	LC651168	DRR328228	Acute pneumonia	Assembling	13,208,520	575,157	14,809.66	5,484	42.12	Monoinfection
Fukushima_H254_2018	LC651169	DRR328229	Acute pneumonia, febrile convulsion	Assembling	13,259,719	781,149	20,516.24	5,561	42.37	Monoinfection
Fukushima_H315_2018	LC651170	DRR328230	Acute bronchitis	Assembling	26,811,533	5,702,012	151,634.54	5,590	42.06	Monoinfection
Fukushima_H565_2019	LC651171	DRR328231	Acute bronchitis	Assembling	8,726,152	272,385	7,244.30	5,596	42.61	Monoinfection
Fukushima_O210_2018	LC651172	DRR328232	Acute epiglottitis	Assembling	30,670,275	689,693	18,204.93	5,450	42.46	Monoinfection
Fukushima_O234_2018	LC651173	DRR328233	Bronchopneumonia	Assembling	27,303,129	45,973	1,211.76	5,291	42.55	Coinfection with adenovirus 2
Fukushima_O278_2018	LC651174	DRR328234	Acute pneumonia	Assembling	14,346,631	2,248,645	59,245.02	5,472	42.27	Monoinfection
Fukushima_OR5_2020	LC651176	DRR328235	Acute pharyngitis	Mapping	9,533,747	3,719	98.18	5,539	42.5	Monoinfection
Fukushima_OR59_2020	LC651177	DRR328236	Bronchial asthma	Assembling	62,190,703	454,271	12,043.63	5,501	42.46	Monoinfection
Fukushima_OR65_2020	LC651178	DRR328237	Pneumonia	Mapping	13,282,899	26,465	677.54	5,421	42.44	Coinfection with rhinovirus
Fukushima_OR72_2020	LC651179	DRR328238	Febrile convulsion	Assembling	10,367,690	834,422	22,050.27	5,490	42.69	Monoinfection
Fukushima_OR189_2021	LC651175	DRR328239	Intussusception	Assembling	8,612,873	725,001	19,253.62	5,402	42.41	Monoinfection

a Coinfection was determined by multiplex real-time PCR assays for respiratory viruses (12).

**FIG 1:**
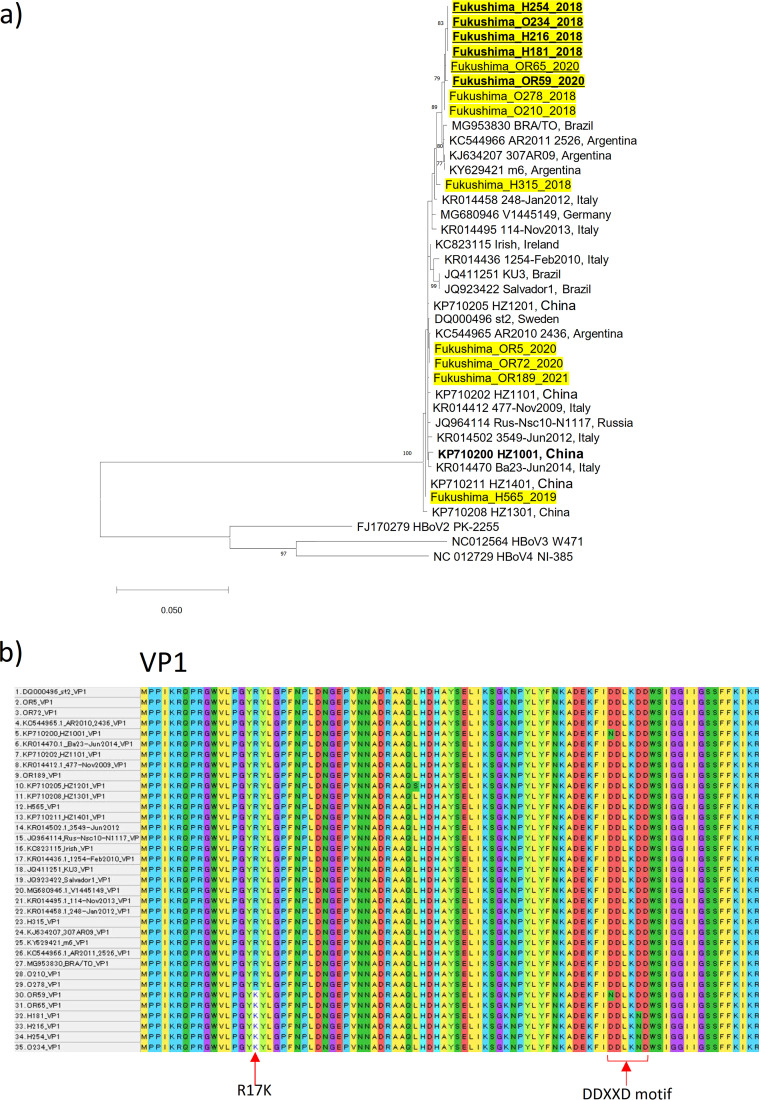
(a) Phylogenetic analysis using the nucleotide sequences of VP1 was performed using MEGA-X software (v10.1.8). The maximum likelihood method was used to generate the phylogenetic tree. Five hundred bootstrap replicates were performed, and only values above 70 are shown. Reference HBoV1 sequences were obtained from GenBank. The new isolates are marked in yellow. The sequences carrying the disrupted DDXXD motif are shown in bold, and strains carrying the R17K amino acid substitution are underlined. The numbers show the bootstrap values. The scale bar shows the number of changes per position. (b) The alignment of VP1 amino acid sequences was prepared using MEGA-X software. Red arrows indicate the positions of amino acid substitutions.

The phylogenetic analysis of the VP1 protein sequences showed that the Fukushima isolates clustered in distinct lineages, closely related to viruses described from around the globe (Fig. 1a). Characteristically, isolates OR59, OR65, H181, H216, H254, and O234 have a substitution at the 17th amino acid in the VP1 protein (R to K) and, except for OR65, also an amino acid substitution in the DDXXD motif (amino acid positions 68 to 72 in VP1), which disrupts this putative metal binding domain (16, 17) (Fig. 1b). Strains carrying both of these amino acid substitutions are not present in databases, suggesting that this unique variant was endemic in Fukushima from 2018 to 2021.

Human subjects were enrolled after approval from the ethics committee of our institute (approval numbers 1001 and 1087).

### Data availability.

The nearly complete genome sequences have been deposited in GenBank under accession numbers LC651167, LC651168, LC651169, LC651170, LC651171, LC651172, LC651173, LC651174, LC651175, LC651176, LC651177, LC651178, and LC651179 (Table 1). The raw reads were deposited under BioProject number PRJDB12572. Run data have been deposited in the DNA Data Bank of Japan (DDBJ) under accession numbers DRR328227, DRR328228, DRR328229, DRR328230, DRR328231, DRR328232, DRR328233, DRR328234, DRR328235, DRR328236, DRR328237, DRR328238, and DRR328239.

## References

[1] Allander T, Tammi MT, Eriksson M, Bjerkner A, Tiveljung-Lindell A, Andersson B. 2005. Cloning of a human parvovirus by molecular screening of respiratory tract samples. Proc Natl Acad Sci USA 102:12891–12896. doi:10.1073/pnas.0504666102. 16118271 PMC1200281

[2] Chen AY, Cheng F, Lou S, Luo Y, Liu Z, Delwart E, Pintel D, Qiu J. 2010. Characterization of the gene expression profile of human bocavirus. Virology 403:145–154. doi:10.1016/j.virol.2010.04.014. 20457462 PMC2879452

[3] Shen W, Deng X, Zou W, Cheng F, Engelhardt JF, Yan Z, Qiu J. 2015. Identification and functional analysis of novel nonstructural proteins of human bocavirus 1. J Virol 89:10097–10109. doi:10.1128/JVI.01374-15. 26223640 PMC4577888

[4] Zou W, Cheng F, Shen W, Engelhardt JF, Yan Z, Qiu J. 2016. Nonstructural protein NP1 of human bocavirus 1 plays a critical role in the expression of viral capsid proteins. J Virol 90:4658–4669. doi:10.1128/JVI.02964-15. 26912614 PMC4836317

[5] Schildgen O, Qiu J, Soderlund-Venermo M. 2012. Genomic features of the human bocaviruses. Future Virol 7:31–39. doi:10.2217/fvl.11.136. 22389649 PMC3291126

[6] Moesker FM, van Kampen JJ, van der Eijk AA, van Rossum AM, de Hoog M, Schutten M, Smits SL, Bodewes R, Osterhaus AD, Fraaij PL. 2015. Human bocavirus infection as a cause of severe acute respiratory tract infection in children. Clin Microbiol Infect 21:964.E1–964.E8. doi:10.1016/j.cmi.2015.06.014. 26100374 PMC7172568

[7] Jiang W, Yin F, Zhou W, Yan Y, Ji W. 2016. Clinical significance of different virus load of human bocavirus in patients with lower respiratory tract infection. Sci Rep 6:20246. doi:10.1038/srep20246. 26832453 PMC4735282

[8] Verbeke V, Reynders M, Flore K, Vandewal W, Debulpaep S, Sauer K, Cardoen F, Padalko E. 2019. Human bocavirus infection in Belgian children with respiratory tract disease. Arch Virol 164:2919–2930. doi:10.1007/s00705-019-04396-6. 31520220 PMC7087345

[9] Huang Q, Deng X, Yan Z, Cheng F, Luo Y, Shen W, Lei-Butters DCM, Chen AY, Li Y, Tang L, Söderlund-Venermo M, Engelhardt JF, Qiu J. 2012. Establishment of a reverse genetics system for studying human bocavirus in human airway epithelia. PLoS Pathog 8:e1002899. doi:10.1371/journal.ppat.1002899. 22956907 PMC3431310

[10] Shen W, Deng X, Zou W, Engelhardt JF, Yan Z, Qiu J. 2016. Analysis of *cis* and *trans* requirements for DNA replication at the right-end hairpin of the human bocavirus 1 genome. J Virol 90:7761–7777. doi:10.1128/JVI.00708-16. 27334591 PMC4988151

[11] Kaida A, Kubo H, Takakura K, Iritani N. 2010. Detection and quantitative analysis of human bocavirus associated with respiratory tract infection in Osaka City, Japan. Microbiol Immunol 54:276–281. doi:10.1111/j.1348-0421.2010.00207.x. 20536724

[12] Kaida A, Kubo H, Takakura K-i, Sekiguchi J-i, Yamamoto SP, Kohdera U, Togawa M, Amo K, Shiomi M, Ohyama M, Goto K, Hase A, Kageyama T, Iritani N. 2014. Associations between co-detected respiratory viruses in children with acute respiratory infections. Jpn J Infect Dis 67:469–475. doi:10.7883/yoken.67.469. 25410563

[13] Shirato K, Kawase M, Matsuyama S. 2018. Wild-type human coronaviruses prefer cell-surface TMPRSS2 to endosomal cathepsins for cell entry. Virology 517:9–15. doi:10.1016/j.virol.2017.11.012. 29217279 PMC7112029

[14] Dijkman R, Koekkoek SM, Molenkamp R, Schildgen O, van der Hoek L. 2009. Human bocavirus can be cultured in differentiated human airway epithelial cells. J Virol 83:7739–7748. doi:10.1128/JVI.00614-09. 19474096 PMC2708629

[15] Shean RC, Makhsous N, Stoddard GD, Lin MJ, Greninger AL. 2019. VAPiD: a lightweight cross-platform viral annotation pipeline and identification tool to facilitate virus genome submissions to NCBI GenBank. BMC Bioinformatics 20:48. doi:10.1186/s12859-019-2606-y. 30674273 PMC6343335

[16] Park J, Rodionov D, De Schutter JW, Lin YS, Tsantrizos YS, Berghuis AM. 2017. Crystallographic and thermodynamic characterization of phenylaminopyridine bisphosphonates binding to human farnesyl pyrophosphate synthase. PLoS One 12:e0186447. doi:10.1371/journal.pone.0186447. 29036218 PMC5643135

[17] Zhang J, Zhang X, Zhang R, Wu C, Guo Y, Mao X, Guo G, Zhang Y, Wang D-C, Li D, Zou Q. 2012. Modeling studies with *Helicobacter pylori* octaprenyl pyrophosphate synthase reveal the enzymatic mechanism of *trans*-prenyltransferases. Int J Biochem Cell Biol 44:2116–2123. doi:10.1016/j.biocel.2012.09.002. 22982238

